# Super-Resolution Quantification of T2DM-Induced Mitochondrial Morphology Changes and Their Implications in Pharmacodynamics of Metformin and Sorafenib

**DOI:** 10.3389/fphar.2022.932116

**Published:** 2022-07-06

**Authors:** Yang Du, Ya-Juan Zhu, Bo Zeng, Xiao-Li Mu, Ji-Yan Liu

**Affiliations:** ^1^ Cancer Center, State Key Laboratory of Biotherapy, Department of Biotherapy, West China Hospital, West China Medical School, Sichuan University, Chengdu, China; ^2^ Dean’s Office, West China Hospital, Sichuan University, Chengdu, China

**Keywords:** mitochondria, structured illumination microscopy, T2DM, metformin, sorafenib

## Abstract

Mitochondria, as the powerhouse of cells, are involved in various processes of cellular homeostasis, especially energy metabolism. The morphology of mitochondria is a critical indicator for their functions, referring to mitochondrial fusion and fission. Here, we performed structured illumination microscopy (SIM) to measure the mitochondrial morphology in living cells. Benefitting from its nano-scale resolution, this SIM-based strategy can quantify the fusion and fission of mitochondria with high sensitivity. Furthermore, as type 2 diabetes mellitus (T2DM) is caused by a disorder of energy substrate utilization, this strategy has the potential to study T2DM by analyzing the mitochondrial morphology of insulin-resistant (IR) cells. With SIM, we found that mitochondrial fission was increased in IR MRC-5, LO2, FHs 74 Int, and HepG2 cells but not in IR Huh7 cells with high-invasiveness ability. Furthermore, we found that metformin could inhibit mitochondrial fission in IR cells, and sorafenib could promote mitochondrial fusion in HepG2 cancer cells, especially in those IR cells. To conclude, mitochondrial fission is involved in T2DM, and cancer cells with high-invasiveness ability may be equipped with stronger resistance to energy metabolism disorder. In addition, the pharmacodynamics of metformin and sorafenib in cancer may be related to the inhibition of mitochondrial fission, especially for patients with T2DM.

## Introduction

Type 2 diabetes mellitus (T2DM), a serious health problem worldwide, is caused by insulin resistance and is related to abnormal energy substrate use in cells (Petersen and Shulman, 2018; Cole and Florez, 2020). Mitochondria play pivotal roles in cellular energy metabolism, and increasing pieces of evidence have pointed out that mitochondrial dysfunction and T2DM are reciprocally influenced (Szendroedi et al., 2012; Prasun, 2020). Thus, studying the role of mitochondria in the pathogenesis of T2DM has attracted more attention from researchers.

Mitochondria are a dynamical organelle, and their morphology can range from tubular networks to sphere structure through fusion and fission events (Scorrano, 2005). Accordingly, as the balance between fusion and fission has direct or indirect effects on mitochondrial function by controlling its quality, content exchange, and ATP production (Mao et al., 2013; Kandul et al., 2016; Chan, 2020), it is increasingly recognized that the function of mitochondria varies with their morphology.

Therefore, expanding our understanding of the changes in mitochondrial morphology is of great significance for T2DM pathogenesis and pharmacodynamics evaluation. To date, the measurement of mitochondrial morphology mainly depended on confocal fluorescence microscopy and transmission electron microscopy (TEM). However, because of the resolution limitation of confocal fluorescence microscopy, it is difficult to sensitively identify and quantify morphological changes in mitochondria in insulin-resistant (IR) cells (Chen et al., 2019). In addition, due to the complicated process, images captured by TEM could rarely reflect the true situation of mitochondrial morphology and cannot be applied in evaluating mitochondria in living cells. Hence, it is essential to develop a more accurate strategy for mitochondrial morphology quantification in living cells. As SIM illuminates the sample with a defined illumination pattern (usually a sine wave grating formed by the interference of two lights) to obtain an image that is beyond the general optical resolution (Gustafsson, 2005), it can capture the morphology of the sample with 100 nm spatial resolution, and has been applied in studying mitochondria and their connections with other organelles in several studies (Chen et al., 2018; Chen et al., 2020; Chen et al., 2021; Wang et al., 2022). But it remains a vacant premise for the SIM-based strategy for mitochondrial morphology in T2DM.

Here, we established IR cells, and performed SIM to capture their mitochondrial morphology at the nano-scale and analysis software to automatically quantify the morphological changes of mitochondria. In addition, to confirm the achieved results and further detect the relationship between T2DM and cancer metastasis, we investigated mitochondrial morphology in IR hepatoma carcinoma cells with different invasive abilities under SIM. Moreover, we measured the changes in mitochondrial morphology in IR cells after being treated with metformin, a classical diabetes drug, or sorafenib, a most-used cancer targeted-therapy drug for hepatocellular carcinoma (HCC), under SIM. Taken together, based on the super-resolution of SIM, we proposed a new strategy for studying T2DM and evaluating the pharmacodynamics of related drugs by quantifying mitochondrial morphology. Also, this method may be a potential tool to guide the therapeutic regimen for T2DM patients with cancers.

## Methods

### Cell Culture

MRC-5, LO2, FHs 74 Int, HepG2, and Huh7 cells were obtained from ATCC and cultured in Dulbecco’s modified Eagle’s medium (DMEM) with 1% penicillin–streptomycin and 10% fetal bovine serum (FBS). All cell lines are cultured under optimal conditions in a humidified atmosphere (95%), 5% CO2 at 37°C. Metformin (Sigma-Aldrich, United States) was dissolved in phosphate-buffered saline (Gibco, United States) as a stock solution of 1M. Sorafenib was purchased from Selleck and dissolved in DMSO (Sigma-Aldrich, United States) as a 50 mM stock solution. For each experiment, fresh appropriate working concentrations were prepared with the DMEM medium. Diluted drugs were filtered through a 0.22-µm PTFE filter (Macherey-Nagel, Germany) before use. MitoTracker^®^ Mitochondrion-Selective Probes were purchased from Invitrogen.

### Cellular Insulin-Resistant Models

To induce insulin resistance, cells were cultured in serum-free, low-glucose (1 g/L) DMEM containing 0.5% BSA and 5 nM of TNF-a (R&D Systems) for 24 h.

### Structured Illumination Microscopy (SIM)-Based Mitochondrial Imaging

Cells were split and cultured in a glass-bottomed micro-well dish and incubated with 2 ml of DMEM supplemented with 10% FBS for 24 h. After being washed with PBS, the cells were treated with metformin (1 mM) or sorafenib (2ug/ml) for 24 h. Afterward, the cells were incubated with 100 nM of MitoTracker Green (MTG) at 37°C for 30 min. Then, the cells were washed with PBS five times and incubated in a phenol-free medium for SIM imaging (Nikon, Tokyo, Japan).

### Statistical Analysis

SIM images were analyzed with Nikon Elements and ImageJ software. Statistical analysis was performed with GraphPad Prism 7.0. The statistical comparison was performed through Student’s t-test at significance levels of *p < 0.05, **p < 0.01, ***p < 0.001, and ****p < 0.0001. Data appeared as mean ± SD.

## Results

Structured Illumination Microscopy (SIM)-Based Imaging of Mitochondrial Morphology for Quantitative Analysis in Insulin-Resistant Cells

**FIGURE 1:**
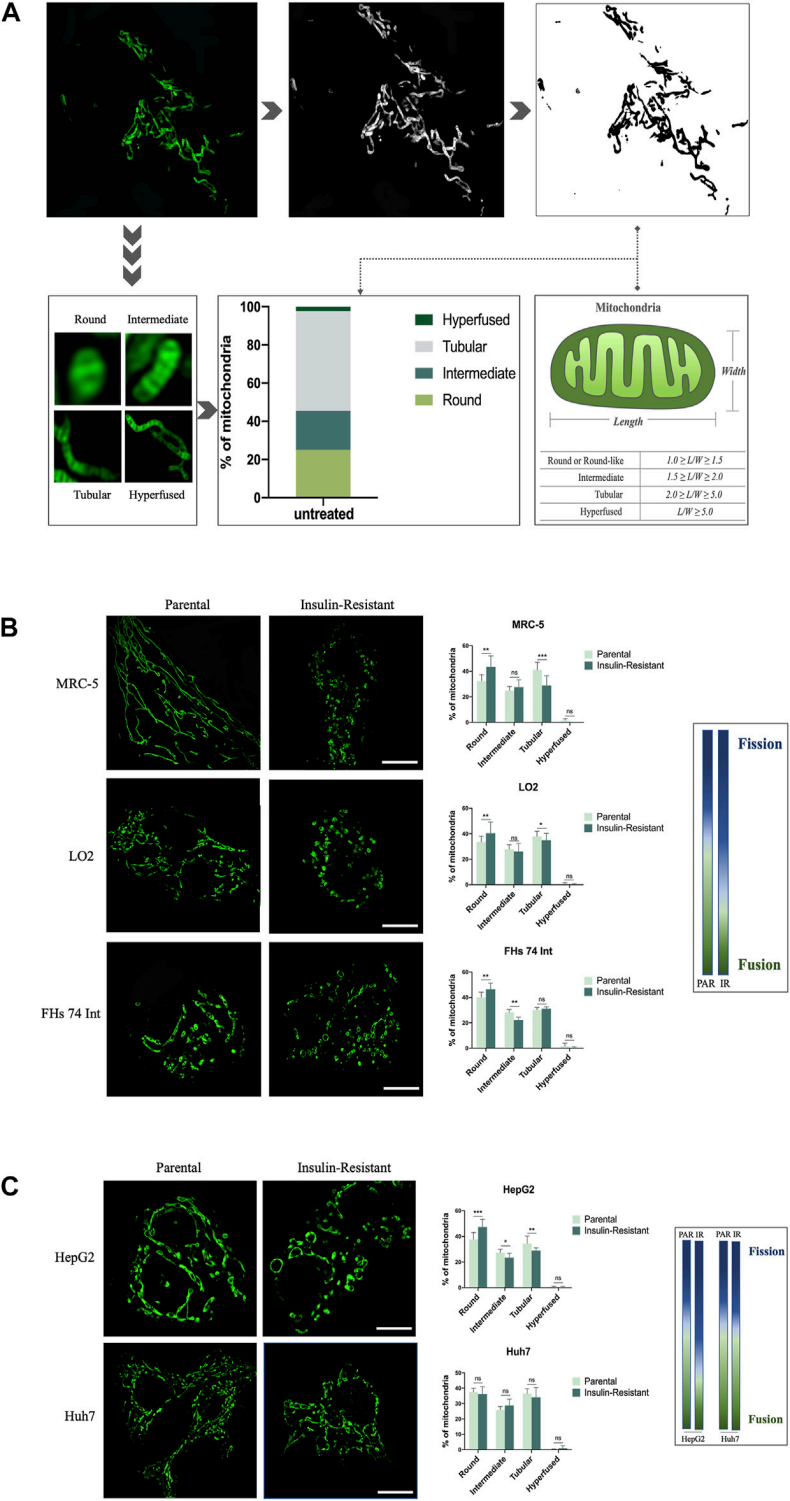
SIM-based imaging of mitochondrial morphology for quantitative analysis in IR cells. **(A)** After being captured by SIM, the image of mitochondria in a 786-O cancer cell was transformed to an 8-bit format, and the length/width of the mitochondria was further analyzed by ImageJ software. **(B)** Representative images of mitochondrial morphology and corresponding quantitative analysis for the four morphological groups (round, intermediate, tubular, and hyper-fused) in parental and IR MRC-5, LO2, and FHs 74 Int cells, respectively. **(C)** Representative images of mitochondrial morphology and corresponding quantitative analysis for the four morphological groups (round, intermediate, tubular, and hyperfused) in parental and IR HepG2 and Huh7 cancer cells, respectively. Scale bar: 10 μm. Data showed as mean ± SD. *p < 0.05, **p < 0.01, and ***p < 0.001, ns: no significance. The gradient column chart indicates the trend of morphological changes, referring to the fission and fusion of the mitochondria.

As the super-resolution of structured illumination microscopy (SIM), which can reach the 100 nm-scale, we combined it with ImageJ software to quantify the mitochondrial morphology in living cells. First, 100 nM MitoTracker Green (MTG) was used to stain 786-O renal cancer cells for 30 min to allow the mitochondria to be captured with SIM. The image showed various morphologies of mitochondria, such as punctate, rod-like, and fibrous. Then, the image was transformed to an 8-bit format by ImageJ software for the next automatical analysis. Next, referring to a previous study (Shao et al., 2020), we used the length-to-width (*L/W*) method to quantify the mitochondrial morphology and assigned it into four classes: round or round-like (1.0 ≤ *L*/*W* < 1.5), intermediate (1.5 ≤ *L*/*W* < 2.0), tubular (2.0 ≤ *L*/*W* < 5.0), and hyper-fused (*L*/*W* ≥ 5.0) (Figure 1A). Thus, we could intuitively observe the distribution of mitochondrial morphology in one cell, and then quantify the morphological changes among cells.

To validate the application of this mitochondrial morphology quantitative strategy based on SIM, and explore the role of morphological changes in mitochondria in T2DM, we first established IR cells with fibroblasts, intestine epithelial cells, and hepatocytes by culturing these three cell lines in a low-glucose medium with TNF-α and 0.5% BSA. This method has been widely used to establish IR cells *in vitro* (Lo et al., 2013). Under SIM, we observed that the round-like mitochondria significantly increased in MRC-5, LO2, and FHs 74 Int cells after they were induced to be resistant to insulin. On the contrary, the tubular mitochondria were decreased, especially in MRC-5 cells. These morphological changes conformed to the mitochondrial fission process. Quantitative analysis results indicated that compared with 32.48 ± 4.77%, 33.51 ± 4.66%, and 40.08 ± 4.06% in parental MRC-5, LO2, and FHs 74 Int cells, respectively, the ratio of round or round-like mitochondria increased to 43.43 ± 8.64%, 40.41 ± 8.73%, and 46.43 ± 4.95% in their corresponding IR cells. Conversely, the ratio of tubular mitochondria decreased to 28.94 ± 7.54% and 34.95 ± 5.36% in IR MRC-5 and LO2 cells, respectively, while they were 41.16 ± 5.85% and 37.87 ± 4.09% in parental MRC-5 and LO2 cells, respectively. The ratio of intermediate mitochondria in FHs 74 Int cells also decreased from 28.24 ± 2.17% to 22.20 ± 2.24% after changing to being insulin-resistant (Figure 1B). Altogether, these data implied that mitochondrial fission is positively correlated with insulin resistance in cells, especially in fibroblasts.

Next, to expand the application of this new strategy in the T2DM area, we continued to measure the mitochondrial morphology in IR cancer cells. We first used HepG2, a hepatoma carcinoma cell line with low-invasive ability, to develop an IR model. We observed that the mitochondria presented as more punctate-like but less fibrosis-like after being induced to be resistant to insulin. Our data also showed the ratio of round or round-like mitochondria increased to 47.34 ± 5.99% in IR HepG2 cells, which was only 37.68 ± 5.39% in its parental cells. Meanwhile, the ratio of intermediate and tubular mitochondria both emerged as opposite changes in IR HepG2 cells compared to their parental cells, decreasing from 27.37 ± 2.56% to 23.43 ± 3.41%, and 34.37 ± 5.93% to 28.99 ± 2.07%, respectively. The balance between fission and fusion tilted to fission. Next, to figure out whether this balance–imbalance is connected with the invasive ability of cancer cells, we induced Huh7 cells, another hepatoma carcinoma cell line but with high-invasive ability, to be resistant to insulin. However, the SIM images showed that there were no obvious changes in mitochondrial morphology after Huh7 cells became insulin-resistant. The data also showed that the distributions of these four morphological classifications in parental Huh7 cells and IR Huh7 cells were similar (Figure 1C). These results implied that the effect of T2DM on cancer may vary from cancer metastatic abilities.

### Decreased Mitochondrial Fission in IR Cells Treated With Metformin or Sorafenib

**FIGURE 2:**
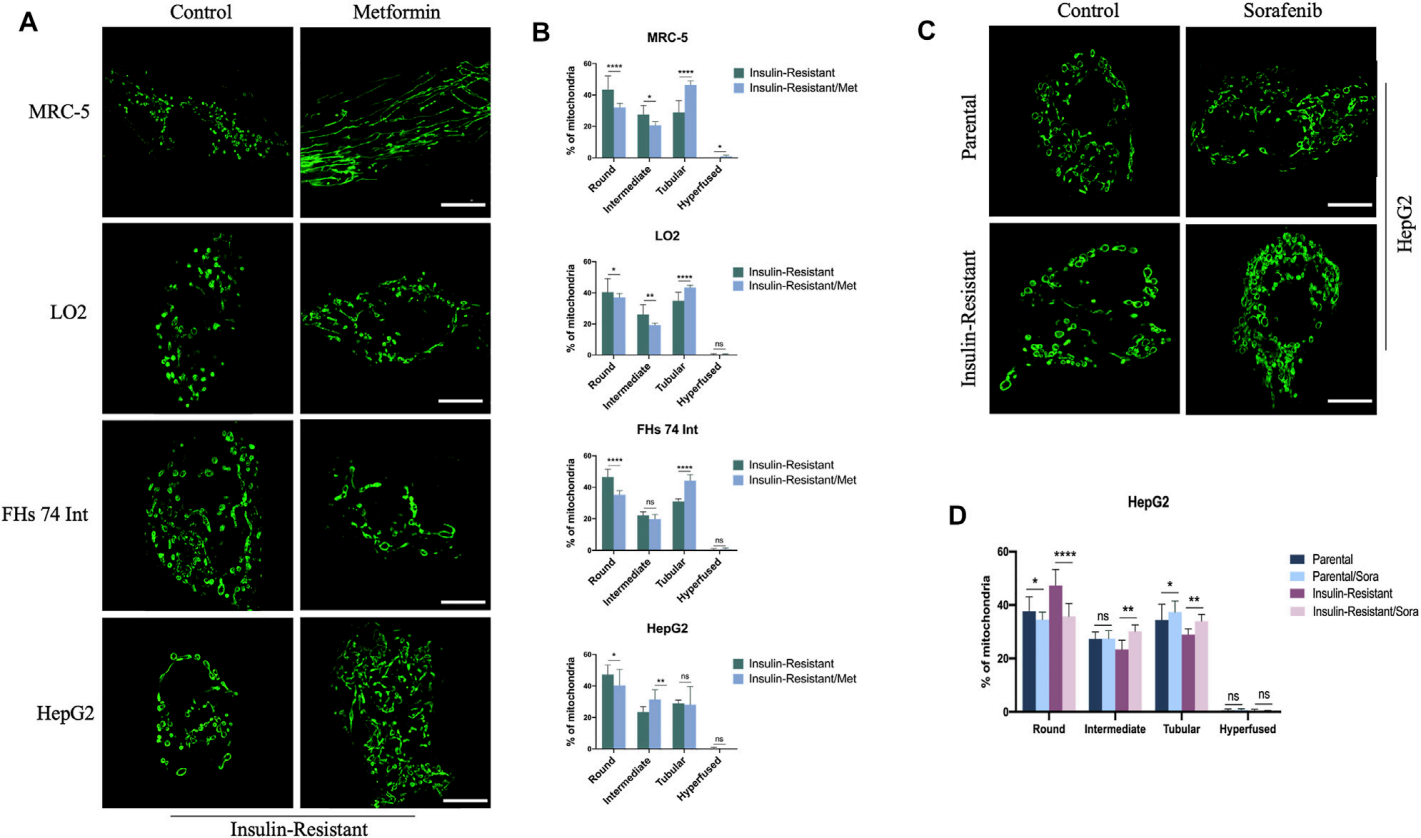
Effect of metformin or sorafenib on mitochondrial morphology in IR cells. **(A)** Representative images of mitochondrial morphology in IR MRC-5, LO2, FHs 74 Int, and HepG2 cells treated or untreated with metformin. **(B)** Quantitative analysis for the four morphological groups (round, intermediate, tubular, and hyper-fused) in IR MRC-5, LO2, FHs 74 Int, and HepG2 cells treated or untreated with metformin. **(C)** Representative images of mitochondrial morphology in IR HepG2 cancer cells with or without sorafenib treatment. **(D)** Quantitative analysis for the four morphological groups (round, intermediate, tubular, and hyper-fused) in IR HepG2 cancer cells with or without sorafenib treatment, respectively. Scale bar: 10 μm. Data showed as mean ± SD. *p < 0.05, **p < 0.01, ***p < 0.001, and ****p < 0.0001, ns: no significance.

As metformin is one of the most used drugs for T2DM therapy, we further evaluated the influence of metformin on the morphological changes of mitochondria in IR cells. With SIM, we observed that the mitochondria exhibited refusion states in the IR MRC-5, LO2, FHs 74 Int, and HepG2 cells after being treated with metformin (Figure 2A). The quantitative data further confirmed this phenomenon. In IR MRC-5 cells, ratios of round or round-like and intermediate mitochondria both slipped from 43.43 ± 8.63% to 32.00 ± 2.72% and from 27.62 ± 5.77% to 20.72 ± 2.24%, respectively, but the ratio of tubular mitochondria increased from 28.94 ± 7.54% to 46.42 ± 2.71%. In IR LO2 cells, ratios of round or round-like and intermediate mitochondria both dropped from 40.41 ± 8.73% to 37.03 ± 2.51% and from 26.09 ± 6.30% to 19.33 ± 1.19%, respectively, but the ratio of tubular mitochondria went up from 34.95% ± 5.36 to 43.31 ± 1.52%. In IR FHs 74 Int cells, the ratios of round or round-like and intermediate mitochondria both dropped from 46.43 ± 4.95% to 35.23 ± 2.52% and from 22.20 ± 2.24% to 19.87 ± 2.91%, respectively, while the ratio of tubular mitochondria increased from 31.08 ± 1.53% to 44.18 ± 3.73%. In HepG2 cancer cells, the ratio of round or round-like mitochondria was also down from 47.34 ± 5.99% to 40.44 ± 10.00%, and the ratio of intermediate mitochondria was up from 23.43 ± 3.41% to 31.38 ± 6.21%; however, the ratio of tubular mitochondria barely changed (Figure 2B). The results gave a hint that metformin might improve insulin resistance in cells by inhibiting their mitochondrial fission, and this refusion effect might also play a role in the anti-cancer effect of metformin.

In addition, as sorafenib is the most commonly used targeted-drug for hepatocellular carcinoma (HCC) therapy, to explore the T2DM influence on sorafenib efficiency for hepatoma carcinoma patients, we proceeded to measure the effect of sorafenib on mitochondrial morphology in parental HepG2 cells and IR HepG2 cells. The images captured by SIM indicated that sorafenib could lead to mitochondria fusion both in these two cells (Figure 2C). With quantitative analysis, the data showed that after treatment with sorafenib, the ratio of round or round-like mitochondria was reduced from 37.68 ± 5.39% to 34.50 ± 2.88% and from 47.34 ± 5.99% to 35.72 ± 4.84%, while the ratio of tubular mitochondria was increased from 34.37 ± 5.93% to 37.32 ± 4.17% and from 28.99 ± 2.07% to 33.97 ± 2.50% in parental and IR HepG2 cells, respectively. Moreover, the ratio of intermediate mitochondria also increased from 23.43 ± 3.41% to 30.12 ± 2.44% in IR HepG2 cells but nearly did not change in parental HepG2 cells (Figure 2D). Overtly, a more distinct fusion trend was detected in IR HepG2 cells, which indicated that sorafenib might be more effective for HCC patients with T2DM.

## Discussion

Increasing amount of evidence has shown that T2DM caused by insulin resistance is characterized by impaired mitochondrial function, and it is also widely accepted that there is an exact link between morphology and function of mitochondria. Thus, studying T2DM from the perspective of mitochondrial morphology will be more intuitional, and it has the potential to provide a quantifiable biomarker for studying and monitoring T2DM. In this study, we used a super-sensitive method, SIM, to examine the morphological changes in mitochondria. The results indicated that the fission of mitochondria was enhanced after normal fibroblasts, hepatocytes, and intestine epithelial cells were induced to be resistant to insulin, especially in fibroblasts. A similar phenomenon has also been reported that the fission of mitochondria can lead to insulin resistance in skeletal muscle cells and dorsal vagal complex (Filippi et al., 2017; Axelrod et al., 2021; Kugler et al., 2021). It should be noted that it is first reported that this phenomenon can be detected in fibroblasts and intestinal epithelial cells in our study. Furthermore, benefitting from the super-sensitivity of SIM, we could accurately quantify the morphological changes of mitochondria with ImageJ software, and the data showed insulin resistance had the greatest influence on fibroblasts among these three cell types. We speculated that the reason for this result is that insulin has a greater effect on mesenchymal cells, such as adipocytes which can be differentiated from fibroblasts *in vitro* (Benito et al., 1991).

In addition, in the past decades, there has been an explosion in knowledge about the role of T2DM in promoting cancer metastasis (Brown et al., 2020; Fan et al., 2020), but the underlying mechanism is still under study. Hence, we further quantified the mitochondrial morphology in IR HepG2 cancer cells and found that mitochondrial fission was also increased. Considering previous research studies which showed that mitochondrial fission was significantly unregulated in distant metastasis of hepatocellular carcinoma and mitochondrial fission can promote breast cancer and hepatocellular carcinoma metastasis (Zhang et al., 2017; Sun et al., 2018; Yu et al., 2021), it is tempting to speculate that T2DM can facilitate cancer metastasis by boosting mitochondrial fission according to our results. Furthermore, we also noted that compared with HepG2 cells of weakly potential invasion and low frequent metastasis, Huh7 cells of strongly potential invasion and high frequent metastasis are less sensitive to insulin resistance; the results suggested that insulin resistance may have a greater effect on the early stage of cancer rather than metastatic cancer. Together, our work added a novel explanation for the metastasis-promoting effect of T2DM in different cancer stages.

As one of the most used first-line drugs for T2DM, metformin also has anticancer effects (Vancura et al., 2018; Cheng et al., 2021; Xiao et al., 2022). Here, our data indicated that metformin could alleviate the mitochondrial fission exerted by insulin resistance in cells. Previous research studies have reported that mitochondrial refusion by the overexpression of Mfn-2, a mitochondrial fusion protein, or inhibition of Drp-1, a mitochondrial fission protein, could lead to a decrease in cell proliferation and an increase in apoptosis in lung cancer (Rehman et al., 2012). It also has been observed that induction of mitochondrial fission is required for the maintenance of liver cancer-initiating cells, and the inhibition of fission could result in the apoptosis of brain tumor-initiating cells; thus, tumor growth was halted (Xie et al., 2015; Tang et al., 2021). Furthermore, mitochondrial fission is important for damaged mitochondrion elimination by mitophagy, so it is possible that the refusion of mitochondria can accumulate damaged mitochondria which go against cancer cells’ survival and proliferation (Grandemange et al., 2009). Given this, we reasoned that the inhibition of mitochondrial fission might be the underlying mechanism of the anticancer effect of metformin. Moreover, we also found that sorafenib could enhance mitochondrial fusion in HepG2 cells, and this effect is stronger in IR HepG2 cells, which is consistent with a previous study which showed that sorafenib might have a better anticancer effect in hepatocellular carcinoma patients with T2DM than those without T2DM (Di Costanzo et al., 2017). Consequently, our data provided a possible explanation for this phenomenon and a new direction to investigate the mechanism for the anti-cancer effect of sorafenib.

By using the super-resolution characteristic of SIM to precisely quantify the morphological changes of mitochondria, we found that insulin resistance could lead to mitochondrial fission in living cells, and metformin and sorafenib can both weaken this mitochondrial fission-promoting effect of insulin resistance. Our work confirmed the viability of the SIM-based strategy in studying T2DM at the nano-scale level. Moreover, the results might provide a new perspective to clarify the mechanism for the effect of T2DM on promoting cancer metastasis and guidelines for medication regimens for cancer patients with T2DM.

## Data Availability

The original contributions presented in the study are included in the article/Supplementary Material; further inquiries can be directed to the corresponding author.
